# *β-catenin* is required in the neural crest and mesencephalon for pituitary gland organogenesis

**DOI:** 10.1186/s12861-016-0118-9

**Published:** 2016-05-16

**Authors:** Shannon W. Davis, Amanda H. Mortensen, Jessica L. Keisler, Amanda L. Zacharias, Philip J. Gage, Ken-ichi Yamamura, Sally A. Camper

**Affiliations:** Department of Biological Sciences, University of South Carolina, 715 Sumter St. CLS room 401, Columbia, SC 29208 USA; Department of Human Genetics, University of Michigan, Ann Arbor, MI 48109 USA; Department of Ophthalmology and Visual Sciences, University of Michigan, Ann Arbor, MI 48105 USA; Institute of Resource Development and Analysis, Kumamoto University, Kumamoto, 860-0811 Japan; Present address: Department of Genetics, University of Pennsylvania, Philadelphia, PA 19104 USA

**Keywords:** Neural crest, Pituitary gland, Organogenesis, *β-catenin*, Vasculature

## Abstract

**Background:**

The pituitary gland is a highly vascularized tissue that requires coordinated interactions between the neural ectoderm, oral ectoderm, and head mesenchyme during development for proper physiological function. The interactions between the neural ectoderm and oral ectoderm, especially the role of the pituitary organizer in shaping the pituitary precursor, Rathke’s pouch, are well described. However, less is known about the role of head mesenchyme in pituitary organogenesis. The head mesenchyme is derived from definitive mesoderm and neural crest, but the relative contributions of these tissues to the mesenchyme adjacent to the pituitary are not known.

**Results:**

We carried out lineage tracing experiments using two neural crest-specific mouse *cre* lines, *Wnt1-cre* and *P0-cre*, and determined that the head mesenchyme rostral to the pituitary gland is neural crest derived. To assess the role of the neural crest in pituitary development we ablated it, using *Wnt1-cre* to delete *Ctnnb1* (*β-catenin),* which is required for neural crest development*.* The *Wnt1-cre* is active in the neural ectoderm, principally in the mesencephalon, but also in the posterior diencephalon. Loss of *β-catenin* in this domain causes a rostral shift in the ventral diencephalon, including the pituitary organizer, resulting in pituitary dysmorphology. The neural crest deficient embryos have abnormally dilated pituitary vasculature due to a loss of neural crest derived pericytes.

**Conclusions:**

*β-catenin* in the *Wnt1* expression domain, including the neural crest, plays a critical role in regulation of pituitary gland growth, development, and vascularization.

**Electronic supplementary material:**

The online version of this article (doi:10.1186/s12861-016-0118-9) contains supplementary material, which is available to authorized users.

## Background

In comparison to invertebrate chordates, vertebrates are characterized by an elaborate head with an expanded brain, elaborate sensory organs, and a craniofacial skeleton. These modifications from the ancestral chordate where enabled in part by the appearance of neural crest cells, neurogenic placodes, and muscularized hypomeres, which are derived from definitive mesoderm, in the ancestral vertebrate [1, 2]. These three tissues, along with the neural ectoderm, interact in diverse ways to form the elaborate head structures of vertebrates, including sensory organs and craniofacial skeletal components [3, 4]. The pituitary gland also appears with the formation of vertebrates and is partially derived from the adenohypophyseal placode [5]. The contributions of the adenohypophyseal placode to pituitary development are well described [5, 6]; however, much less is known about neural crest and definitive mesoderm contributions to pituitary organogenesis.

Rathke’s pouch, the precursor to the pituitary anterior and intermediate lobes, is surrounded by cranial head mesenchyme and is in close proximity to the rostral end of the notochord and pre-chordal plate, which have important signaling functions in early head development [7]. Chick explant studies demonstrated that co-cultures of the ventral diencephalon and Rathke’s pouch could only induce differentiation of corticotropes when mesenchyme was included in the culture [8]. Additionally, explants of chick notochord can cause surface ectoderm to invaginate, forming a structure similar to Rathke’s pouch [8]. These experiments suggest that chick head mesenchyme plays a role in supporting cell differentiation in the anterior lobe. The permissive mesenchymal signal for anterior lobe cell specification has not been identified. Several secreted factors expressed in mouse pituitary adjacent mesenchyme, including *Chordin*, *Noggin*, *Nbl1*, and *Fstl1,* are candidates for a mesenchymal signal in pituitary organogenesis [9–11]. *Foxd1* is a forkhead domain transcription factor expressed in the pituitary adjacent mesenchyme, and *Foxd1*^*−/−*^ mice have increased proliferation of anterior lobe cells and decreased LHβ expression. These results suggest that *Foxd1* may regulate the expression of a mesenchymal signal necessary for pituitary development [12].

The cranial mesenchyme contributes to the hypophyseal portal system, which is a network of blood vessels that surrounds and invades the pituitary gland, enabling delivery of releasing hormones from the hypothalamus to the pituitary anterior lobe and from the pituitary gland to target organs in the body. The head mesenchyme is comprised of both definitive mesoderm, migrating through the primitive streak, and neural crest, which migrates away from the dorsal side of the neural tube and throughout the body [4]. The neural crest forms much of the peripheral nervous system, which is consistent with its ectodermal origin, but within the head it contributes to tissues, such as bones, muscles, and dermis of the skin, which are usually derived from definitive mesoderm [13]. In recognition of the diversity of tissues generated by the neural crest, it is frequently described as a fourth germ layer [14]. The developmental plasticity of the neural crest results from the maintenance of a gene regulatory network, characteristic of pluripotent blastula cells, in the neural crest lineage [15]. The cranial vasculature reflects the dual contributions of definitive mesoderm and neural crest; endothelial cells are derived from definitive mesoderm and the neural crest forms the pericytes and smooth muscle that wrap around and regulate the endothelial cells [16–18]. To define the contributions of head mesenchyme to pituitary organogenesis, we utilized a genetic model to examine neural crest contributions to pituitary gland organogenesis. β-CATENIN is a key component of canonical Wnt signaling, and loss of *β-catenin* in the neural crest lineage is known to cause apoptosis of migrating neural crest cells, leading to severe head malformations [19]. We present here an analysis of pituitary gland organogenesis in the absence of neural crest, and uncovered a critical role for neural crest contributions to the pituitary vasculature.

## Results

**Fig. 1:**
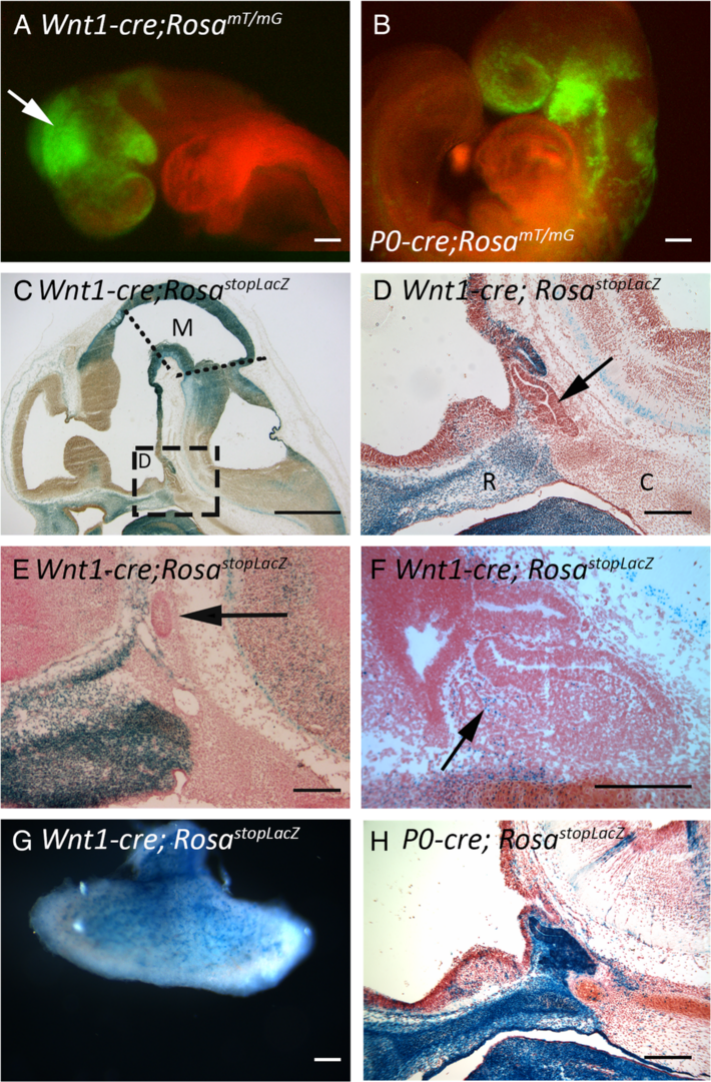
**a**–**b** *Rosa* ^*mT/mG*^ reporter mice enable fluorescent detection of cre recombinase activity, where green fluorescence indicates areas of cre recombination and red fluorescence indicates areas with no recombination. **a** *Wnt1-cre; Rosa* ^*mT/mG*^ e8.5 embryo. Arrow indicates recombinase activity in the midbrain. **b** *P0-cre; Rosa* ^*mT/mG*^ e8.5 embryo. **c**–**h** X-gal staining (blue) reveals *cre* recombinase activity in *Rosa* ^*stopLacZ*^ reporter mice. **c**–**f** and **h** sagittal sections counterstained with neutral red. **c** Mid-sagittal section of an e12.5 *Wnt1-cre; Rosa* ^*stopLacZ*^embryo. Dotted lines indicate the midbrain (M) boundaries with the diencephalon and hindbrain. The boxed area is magnified in **d**. **d** Boxed area indicated in **c**. Arrow indicates Rathke’s pouch; R indicates rostral head mesenchyme; C indicates caudal head mesenchyme **e** e12.5 *Wnt1-cre; Rosa* ^*stopLacZ*^ section at the lateral extreme of Rathke’s pouch (arrow). **f** e14.5 *Wnt1-cre; Rosa* ^*stopLacZ*^. Arrow indicates X-gal stained cells within the pituitary anterior lobe. **g** Ventral view of a dissected e18.5 *Wnt1-cre; Rosa* ^*stopLacZ*^ pituitary. **h** Mid-sagittal section of an e12.5 *P0-cre; Rosa* ^*stopLacZ*^ embryo. Scale bars in **a**, **b**, and **d**–**h** equal 100 μm. Scale bar in **c** equals 1 mm

*Tg(Wnt1-cre)11Rth* (abbreviated here as *Wnt1-cre*) and *C57BL/6 J-Tg(P0-Cre)94Imeg* (*P0-cre*) are frequently used to fate map and generate tissue specific loss-of-function alleles in the neural crest [20, 21]. To determine the contributions of the neural crest to head mesenchyme near the developing pituitary gland we crossed both the *Wnt1-cre* and *P0-cre* lines with the reporter lines B6.*Gt(ROSA)26Sor*^*tm4(ACTB-tdTomato,-EGFP)Luo*^ (*Rosa*^*mT/mG*^) and *Gt(ROSA)26Sor*^*tm1Sho*^ (*Rosa*^*stopLacZ*^) to determine neural crest contributions to the pituitary adjacent head mesenchyme [22, 23]. At embryonic day of development 8.5 (e8.5) both *Wnt1-cre* and *P0-cre* mediate recombination in the migrating neural crest cells (Fig. 1a and b). The *Wnt1-cre* also mediates recombination in the midbrain, a domain of *cre* activity that is not observed in the *P0-cre*. The midbrain activity of *Wnt1-cre* is also observed at e12.5, but it is not confined to the midbrain, as *LacZ* is expressed in the posterior diencephalon and in the anterior hindbrain (Fig. 1c). At e12.5 the head mesenchyme rostral to Rathke’s pouch expresses *LacZ*, indicating that it is derived from the neural crest (Fig. 1d and e). The head mesenchyme caudal to Rathke’s pouch is devoid of X-gal staining, suggesting that this tissue is derived from definitive mesoderm [7, 24, 25]. A sharp boundary between the neural crest derived head mesenchyme and the definitive mesenchyme is observed ventral to Rathke’s pouch in midsagittal sections (Fig. 1d). Rathke’s pouch is located at the rostral end of the notochord, and chick explants studies demonstrate that the notochord may be involved in Rathke’s pouch formation [8, 26, 27]. At more lateral sections the boundary between neural crest and definitive mesoderm is not as sharp, as the definitive mesoderm extends past the lateral edge of Rathke’s pouch (Fig. 1e). At e14.5 X-gal stained cells are detected in the forming pituitary anterior lobe of *Wnt1-cre; Rosa*^*stopLacZ*^ embryos, suggesting that the neural crest derived mesenchyme is invading the oral ectoderm-derived Rathke’s pouch tissue (Fig. 1f). By e18.5 *Wnt1-cre* expressing cells have made a significant contribution to the pituitary gland (Fig. 1g). Lineage tracing in *P0-cre; Rosa*^*stopLacZ*^ embryos at e12.5 also demonstrates a sharp boundary between neural crest and definitive mesoderm at mid-sagittal locations (Fig. 1h). Although *Wnt1-cre* activity in the midbrain and neural crest and *P0-cre* activity in the neural crest mimic endogenous *Wnt1* and *P0* expression, we also observed ectopic activity in the ventral diencephalon for both *cre* lines (Fig. 1d and h), and *cre* activity in Rathke’s pouch for the *P0-cre* (Fig. 1h).

**Fig. 2:**
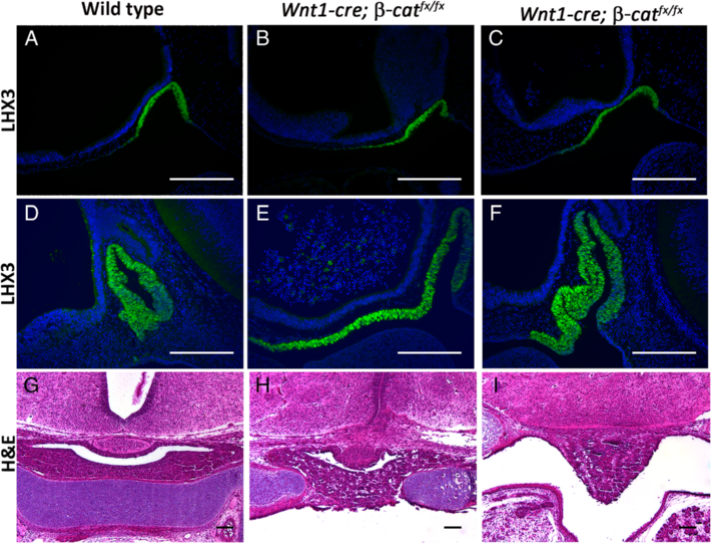
**a** – **f** LHX3 immunostaining (green) on sagittal sections, counterstained with DAPI (blue). **a** Wild type e10.5 **b** & **c** Two separate *Wnt1-cre; β-cat* ^*fx/fx*^ e10.5 embryos **d** Wild type e12.5 **e** & **f** Two separate *Wnt1-cre; β-cat* ^*fx/fx*^ e12.5 embryos **g** – **i** Hemotoxylin and eosin staining on e18.5 coronal sections **g** Wild type **h** & **i** Two sections from the same *Wnt1-cre; β-cat* ^*fx/fx*^ embryo, with I representing a more rostral location. All scale bars equal 100 μm

Previous work established that *Wnt1-cre* mediated deletion of *β-catenin* causes neural crest cells to undergo apoptosis during migration, which provides a method to generate neural crest deficient embryos [19]. Because pituitary development was not analyzed in these embryos, we crossed *Wnt1-cre* with *B6.129-Ctnnb1*^*tm2Kem*^*/KnwJ* (referred to here as *β-cat*^*fx/fx*^) and then generated *Wnt1-cre;β-cat*^*fx/fx*^ embryos to determine if the neural crest derived head mesenchyme is important for pituitary organogenesis. Indeed, *Wnt1-cre;β-cat*^*fx/fx*^ embryos do have abnormalities in pituitary development (Fig. 2a-i). At early stages of Rathke’s pouch formation (e10.5) a larger domain of oral ectoderm is recruited into Rathke’s pouch, as marked by immunostaining for the critical LIM homeodomain transcription factor, LHX3 (Fig. 2b and c). At e12.5 the enlarged, mutant Rathke’s pouch includes more oral ectoderm tissue than typical, extending more rostrally than normal (Fig. 1e). The enlarged domain of presumptive pituitary tissue becomes highly dysmorphic (Fig. 2f). The abnormal pituitary extends through the cartilage plate that underlies the pituitary gland at e18.5 and can form an anterior lobe like structure that projects into the oral cavity at more rostral locations (Fig. 2h and i).

**Fig. 3:**
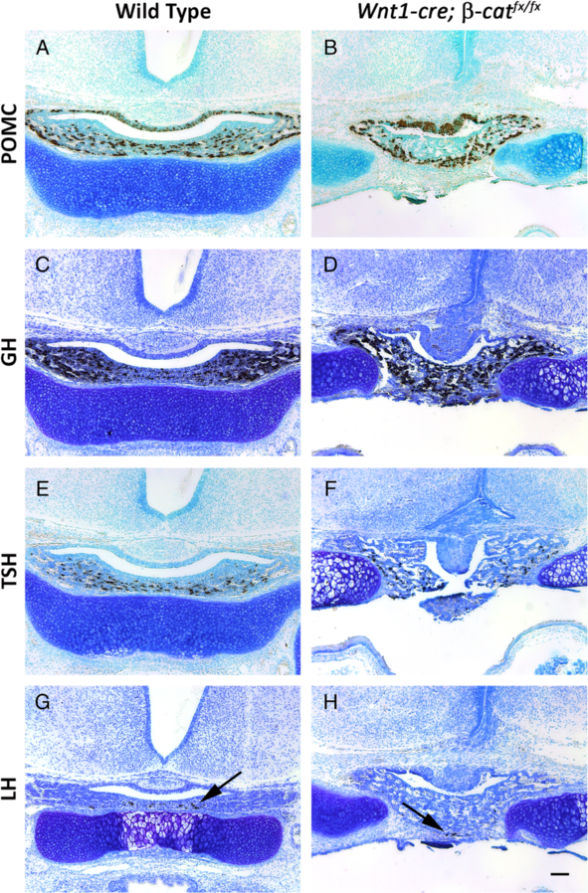
Immunostaining for pituitary anterior and intermediate lobe hormones (brown) at e17.5, counterstained with methyl green (blue). **a** & **b** Proopiomelanocortin (POMC) **c** & **d** Growth hormone (GH) **e** & **f** Thyroid stimulating hormone (TSH) **g** & **h** Luteinizing hormone (LH), arrows indicate select positive cells. **a**, **c**, **e**, & **g** Wild type **b**, **d**, **f**, & **h** *Wnt1-cre; β-cat* ^*fx/fx*^. Scale bar in H equals 100 μm for all images

The number and distribution of hormone expressing cell types in the pituitary anterior lobe in both wild type and *Wnt1cre; β-cat*^*fx/fx*^ embryos was examined by immunostaining for pituitary hormones at e18.5 (Fig. 3a–h). No obvious difference in the quantity or distribution of anterior lobe cell types was detected between the wild type and mutant embryos, despite the highly dysmorphic, mutant pituitary gland.

**Fig. 4:**
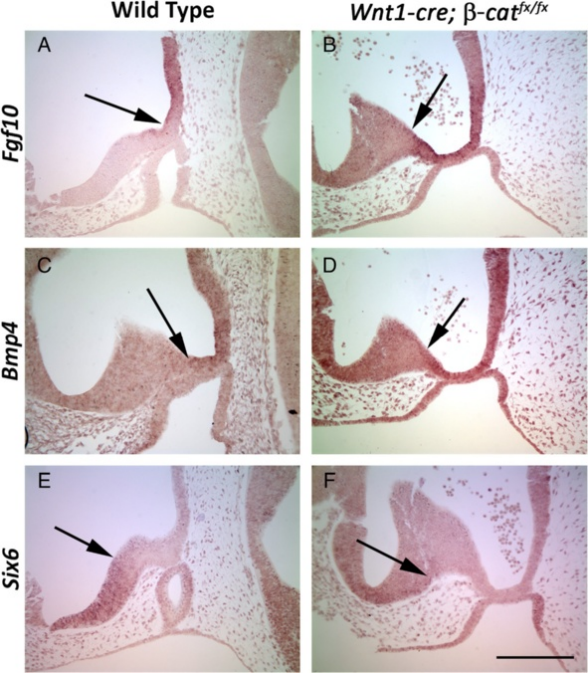
RNA in situ hybridization on e10.5 sagittal sections (brown). Arrows represent the boundary of expression between the domain of *Bmp4* and *Fgf10* expression and adjacent *Six6* expressing domain in the ventral diencephalon. **a** & **b** *Fgf10* **c** & **d** *Bmp4* **e** & **f** *Six6* **a**, **c**, & **e** Wild type **b**, **d**, & **f** *Wnt1-cre; β-cat* ^*fx/fx*^. Scale bar in F equals 100 μm for all images

The dysmorphic pituitary of *Wnt1cre; β-cat*^*fx/fx*^ embryos resembles those of other genetically modified mouse embryos that have an expansion of the pituitary organizer in the ventral diencephalon [6]. The pituitary organizer is characterized in part by the expression of *Fgf10* and *Bmp4*, which are necessary for the induction and proliferation of Rathke’s pouch [28, 29]. We examined *Bmp4* and *Fgf10* expression in wild type and *Wnt1cre; β-cat*^*fx/fx*^ embryos by in situ hybridization and found that the mutant embryos have expanded expression domains of both morphogenetic proteins (Fig. 4a – d). The expression domain of the transcription factor SIX6 typically exists in the ventral diencephalon rostral to the pituitary organizer. *Six6* expression is shifted rostrally from Rathke’s pouch in the mutant embryos (Fig. 4e – f). These results demonstrate that the patterning within the ventral diencephalon is disrupted in *Wnt1cre; β-cat*^*fx/fx*^ embryos. Excess BMP and FGF signaling from the pituitary organizer likely contributes to the recruitment of additional oral ectoderm into Rathke’s pouch [9, 30–32].

**Fig. 5:**
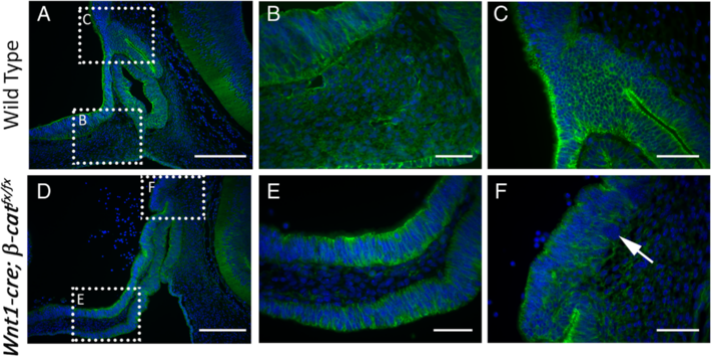
Immunostaining for β-CATENIN (green) on e14.5 sagittal sections, counterstained with DAPI (blue) **a**–**c** Wild type, boxed regions in **a** are magnified in **b** and **c**. **b** Region highlighting neural crest derived mesenchyme **c** Region highlighting ventral diencephalon **d**–**f** *Wnt1-cre; β-cat* ^*fx/fx*^, boxed regions in **d** are magnified in **e** and **f**. **e** Region highlighting neural crest derived mesenchyme where β-CATENIN is lost **f** Region highlighting ventral diencephalon where b-catenin is maintained. Arrow indicates a small region with no β-CATENIN expression. Scale bars in **a** and **d** equal 100 μm, scale bars in **b**, **d**, **e**, and **f** equal 20 μm

*Wnt1-cre* is expected to drive recombination in multiple tissues where loss of *β-catenin* could affect pituitary organogenesis, including the neural crest and ventral diencephalon. We examined β-CATENIN expression by immunohistochemistry to determine the efficiency of *cre* recombination in these tissues (Fig. 5a-f). At e12.5 the *Wnt1-cre; β-cat*^*fx/fx*^ embryos do not express β-CATENIN in the residual neural crest derived mesenchyme rostral to Rathke’s pouch, indicating that *Wnt1-cre* efficiently recombines the conditional *β-catenin* allele in this tissue (Fig. 5d and e). *Wnt1-cre* is not uniformly active within the ventral diencephalon, and only small clumps of cells exhibit loss of β-CATENIN (Fig. 5d and f). This level of *β-catenin* ablation is unlikely to alter ventral diencephalon patterning. However, we may be underestimating the degree of *β-catenin* ablation as the protein persists in the adherens junctions for many hours following recombination at the *β-catenin* locus [33]. In addition, the presence of β-CATENIN protein in the adherens junctions does not maintain canonical Wnt signaling after *cre* mediated recombination [33]. Therefore, we cannot rule out the possibility that *β-catenin* ablation in the infundibular domain may result in an expansion of the pituitary organizer.

**Fig. 6:**
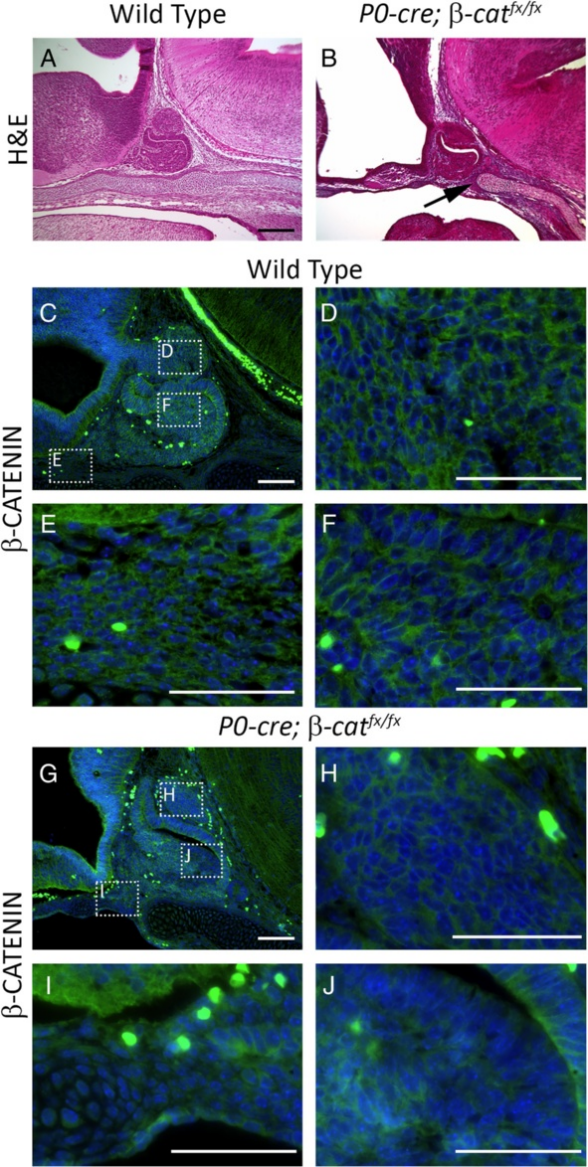
**a** & **b** Hemotoxylin and eosin staining on e14.5 sagittal sections **a** Wild type **b** *P0-cre; β-cat* ^*fx/fx*^, arrow indicates the end of cartilage plate that will form the sphenoid bone. **c**–**j** Immunostaining for β-CATENIN (green) on e14.5 sagittal sections, counterstained with DAPI (blue) **c**–**f** Wild type, boxed regions in **c** are magnified in **d**, **e**, and **f**. **d** Region highlighting the ventral diencephalon **e** Region highlighting the neural crest derived mesenchyme. **f** Region highlighting the pituitary anterior lobe. **g**–**j** *P0-cre; β-cat* ^*fx/fx*^, boxed regions in **g** are magnified in **h**, **i**, and **j**. **h** Region highlighting the ventral diencephalon. **i** Region highlighting the neural crest derived mesenchyme. **j** Region highlighting the pituitary anterior lobe. Scale bars equal 100 μm for **a**–**c** and **g**. Scale bars equal 50 μm for **d**–**f** and **h**–**j**

We sought to recapitulate the *Wnt1-cre; β-cat*^*fx/fx*^ phenotype using the *P0-cre,* which is also active in the neural crest and has some ectopic activity in the ventral diencephalon, to confirm that loss of *β-catenin* in these domains causes the pituitary phenotype. *P0-cre; β-cat*^*fx/fx*^ embryos have severe craniofacial malformations compared to wild type littermates consistent with a neural crest deficiency (Additional file 1: Figure S1), and similar in phenotype to *Wnt1-cre; β-cat*^*fx/fx*^ embryos [19]. However, unlike *Wnt1-cre; β-cat*^*fx/fx*^ embryos, the pituitary morphology of *P0-cre; β-cat*^*fx/fx*^ mutant embryos is very similar to wild type despite the absence of neural crest derived cranial mesenchyme rostral to Rathke’s pouch, as evidenced by the absence of the cartilage plate rostral to the pituitary gland (Fig. 6a and b). Using immunostaining we determined that the *P0-cre* efficiently recombines the conditional *β-catenin* allele in the neural crest, but not in the ventral diencephalon at e12.5 (Fig. 6g–i). Within Rathke’s pouch, variable, small patches of β-CATENIN deficient cells are observed, but β-CATENIN is not completely eliminated (Fig. 6j). Thus, both *Wnt1-cre* and *P0-cre* are similarly effective in deleting *β-catenin* in the neural crest, but the *P0-cre* does not delete *β-catenin* in the ventral diencephalon. Therefore, the *Wnt1-cre; β-cat*^*fx/fx*^ dysmorphic pituitary is not caused by the loss of neural crest cells.

**Fig. 7:**
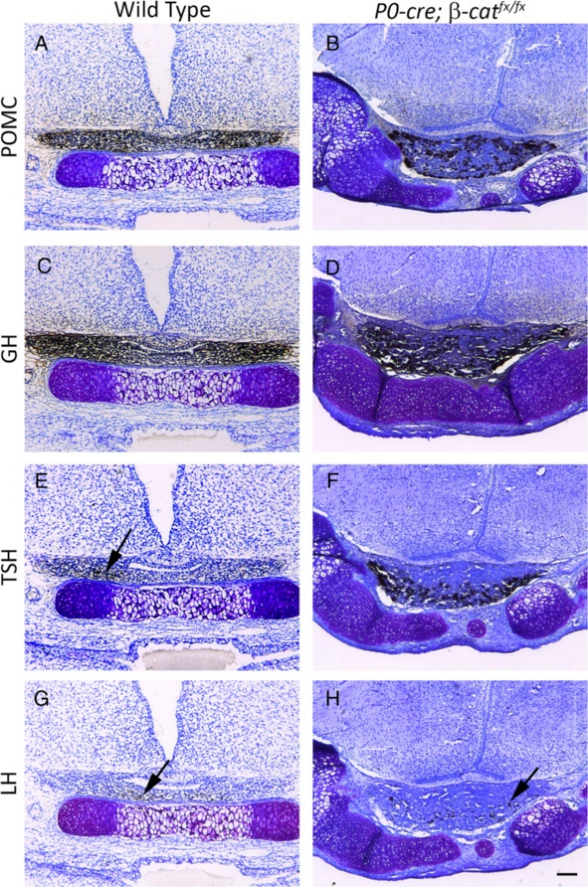
**a** – **h** Immunostaining for pituitary anterior and intermediate lobe hormones (brown) at e17.5, counterstained with methyl green (blue). **a** & **b** POMC **c** & **d** GH **e** & **f** TSH **g** & **h** LH. **a**, **c**, **e**, & **g** Wild type **b**, **d**, **f**, & **h** *P0-cre; β-cat* ^*fx/fx*^. Arrows in **e**, **g**, and **h** indicate positively stained cells. Scale bars equal 100 μm for all images

The *P0-cre* can drive recombination in Rathke’s pouch, and β-CATENIN can influence the activity of the transcription factors PROP1, PIT1 (POU1F1), PITX2, and SF1 (NR5A1) in the pouch and its derivatives [34–37]. Therefore, we sought to determine if the ectopic, mosaic loss of β-CATENIN in the *P0-cre; β-cat*^*fx/fx*^ pouch could alter anterior lobe cell specification. No significant changes in hormone expressing cell types were observed in the mutant embryos (Fig. 7a – h), indicating that the mosaic loss of β-CATENIN in Rathke’s pouch does not disrupt pituitary cell specification.

Wnt signaling in the ventral diencephalon is known to regulate the expression domain of the pituitary organizer [30, 31]. While the mosaic loss of *β-catenin* in the ventral diencephalon observed with *Wnt1-cre* is unlikely to alter pituitary organizer activity, we sought to inactivate *β-catenin* in the ventral diencephalon to determine if canonical Wnt signaling mediates pituitary organizer expression. We crossed *Shh*^*tm1(EGFP/cre)Cjt*^ (*Shh-cre*) with *Rosa*^*stopLacZ*^ and stained embryo sections with X-gal. We detected staining in the ventral diencephalon and the oral ectoderm at e10.5 in a pattern that recapitulates the endogenous expression of *Shh* (Additional file 2: Figure S2a) [38]. We next crossed the *Shh-cre* with *β-cat*^*fx/fx*^ to determine if the loss of *β-catenin* in the ventral diencephalon would lead to an expanded pituitary organizer and an expansion of Rathke’s pouch. At e12.5 the pituitaries of *Shh-cre; β-cat*^*fx/fx*^ embryos were indistinguishable from wild type littermates (Additional file 2: Figure S2b–c). The lack of *β-catenin* in the *Shh* expressing domain of the fore and hind limbs leads to disruptions in digit formation, as expected (Additional file 2: Figure S2d–f). Previous studies have demonstrated that *β-catenin* is necessary for maintaining limb mesenchyme and *Fgf10* expression in the apical ectodermal ridge [39]. We used immunostaining to determine whether the *Shh-cre* effectively deleted β-CATENIN in the ventral diencephalon. Although there were patches of cells that were negative for β-CATENIN immunostaining, the majority of cells in the ventral diencephalon retained β-CATENIN expression (Additional file 2: Figure S2g–i). We concluded that unlike in the limb bud, the *Shh-cre* is unable to efficiently recombine the *β-cat*^*fx/fx*^ allele in the ventral diencephalon, leaving canonical Wnt signaling largely intact. This result also confirms that the mosaic loss of *β-catenin* in the ventral diencephalon is insufficient for producing the dysmorphic pituitaries of *Wnt1-cre; β-cat*^*fx/fx*^ embryos.

There are many precedents for variability in *cre*-mediated deletion, including parent-of-origin effects, episome formation, number of conditional alleles, and genetic background differences, any of which could affect the activity of the transgenic *cre* lines used in this study [40–43]. However, the three *cre* lines used here demonstrate that *β-catenin* deletion in the neural crest and mosaic *β-catenin* deletion in the ventral diencephalon do not generate a dysmorphic pituitary gland. The major difference in activity between the *cre* lines is the expression of *Wnt1-cre* in the midbrain.

**Fig. 8:**
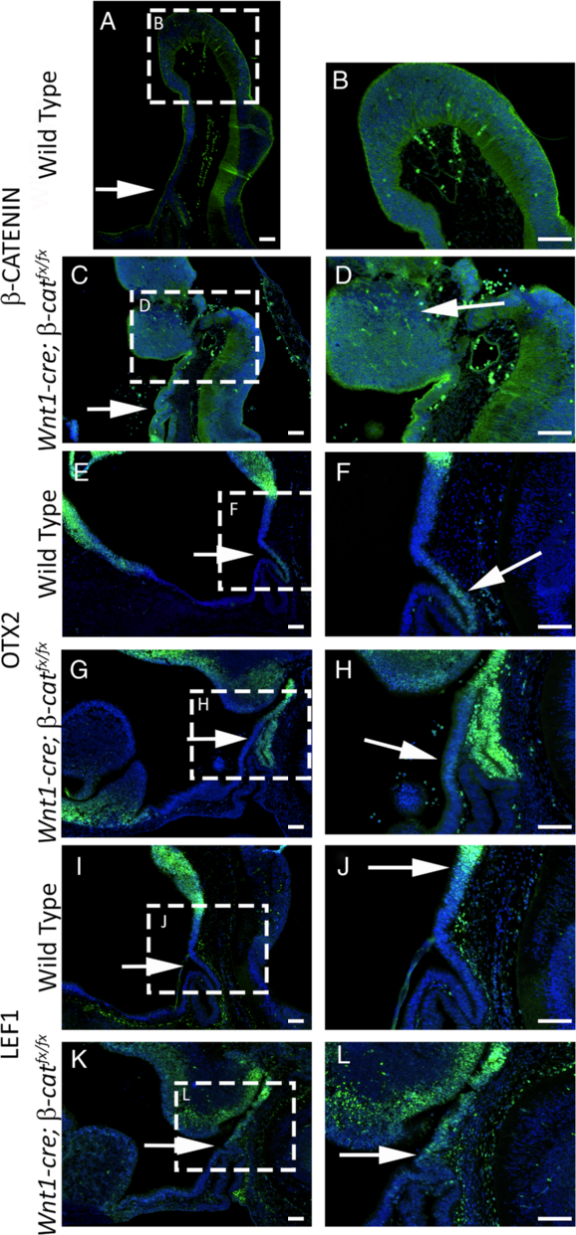
**a** – **d** Immunostaining for β-CATENIN (green) on e11.5 sagittal sections, counterstained with DAPI (blue). **a** and **b** Wild type embryo. Arrow in **a** indicates the level of the infundibulum. Boxed region is magnified in **b**. **c** and **d** *Wnt1-cre; β-cat* ^*fx/fx*^ embryo. Arrow in **c** indicates the level of the infundibulum. Boxed region is magnified in **d**. Arrow in **d** indicates cells at the posterior diencephalon and midbrain boarder deficient for β-CATENIN. **e** – **h** Immunostaining for OTX2 (green) on e11.5 sagittal sections, counterstained with DAPI (blue). **e** and **f** Wild type embryo. Arrow in **e** indicates the level of the infundibulum. Boxed region is magnified in **f**. Arrow in **f** indicates a low level of OTX2 expression. **g** and **h** *Wnt1-cre; β-cat* ^*fx/fx*^ embryo. Arrow in **g** indicates the level of the infundibulum. Boxed region is magnified in **h**. Arrow in **h** indicates a low level of OTX2 expression. **i** – **l** Immunostaining for LEF1 (green) on e11.5 sagittal sections, counterstained with DAPI (blue). (**i** and **j**) Wild type embryo. Arrow in **i** indicates the level of the infundibulum. Boxed region is magnified in **j**. Arrow in **j** indicates the boundary of LEF1 expression. **k** and **L** *Wnt1-cre; β-cat* ^*fx/fx*^ embryo. Arrow in **k** indicates the level of the infundibulum. Boxed region is magnified in **l**. Arrow in L indicates the boundary of LEF1 expression. All scale bars equal 100 μm

We tested the possibility that loss of *β-catenin* in the midbrain results in patterning disruptions of the ventral diencephalon by examining additional markers of the posterior ventral diencephalon. Our fate mapping studies suggest that the *Wnt1-cre* is also active in the posterior diencephalon (Fig. 1c). Immunoflourescence for β-CATENIN on e11.5 wild type and *Wnt1-cre; β-catenin*^*fx/fx*^ embryos demonstrates that β-CATENIN expression is lost in many cells near the boundary of the midbrain and posterior diencephalon (Fig. 8a–d). *Otx2* is expressed in the forebrain and midbrain and is necessary for forming the isthmic organizer at the midbrain/hindbrain boundary [44–46]. During Rathke’s pouch induction, OTX2 is expressed in the infundibulum and more posterior regions of the ventral diencephalon, but is excluded from the neural ectoderm rostral to the infundibulum [47]. At e11.5 OTX2 is strongly expressed in the posterior diencephalon, but has a lower level of expression in the infundibulum (Fig. 1e and f). In *Wnt1-cre; β-cat*^*fx/fx*^ embryos OTX2 is strongly expressed in the infundibulum and a domain of weaker OTX2 expression is observed rostral to the infundibulum (Fig. 8g and h). *Lef1* is expressed in the premamillary region of the developing hypothalamus [48]. This domain of LEF1 expression is not contiguous with the infundibulum (Fig. 8i and j). In *Wnt1-cre; β-cat*^*fx/fx*^ embryos the boundary of LEF1 expression is displaced rostrally and corresponds to the caudal edge of the infundibulum (Fig. 8k and l). These results demonstrate that the patterning of the entire posterior ventral diencephalon, including the pituitary organizer, is shifted rostrally when *β-catenin* is deleted by the *Wnt1-cre*.

**Fig. 9:**
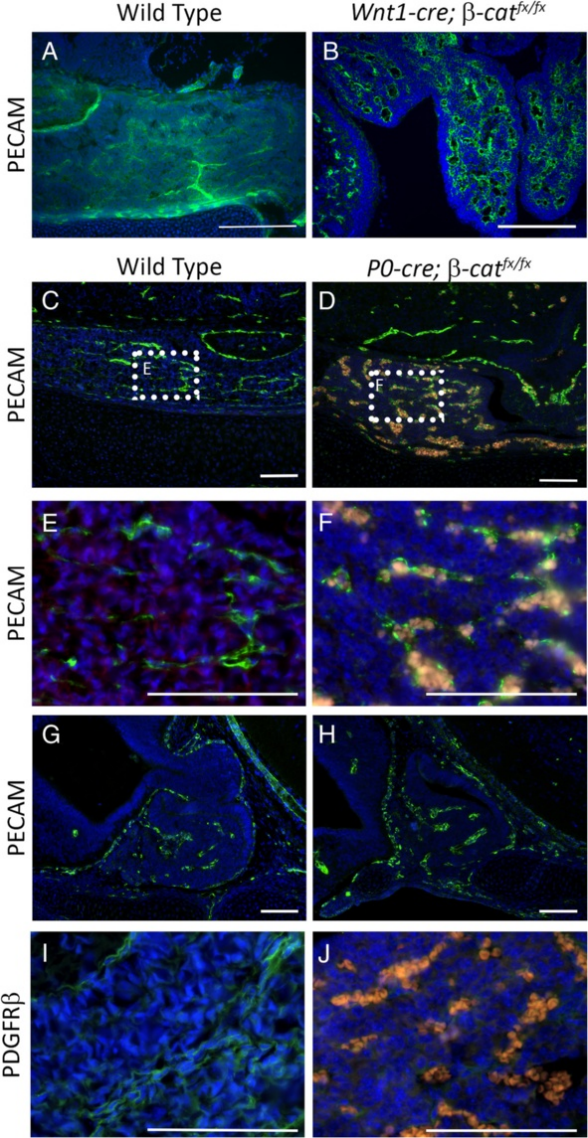
**a** – **h** Immunostaining for PECAM (green), counterstained with DAPI (blue). **a** Wild type e17.5 cryosection, coronal orientation **b** *Wnt1-cre; β-cat* ^*fx/fx*^ e17.5 cryosection, coronal orientation **c** & **e** Wild type e18.5 paraffin section, coronal orientation. Boxed area in **c** is magnified in **e**. **d** & **f** *P0-cre; β-cat* ^*fx/fx*^ e18.5 paraffin section, coronal orientation. Boxed area in **d** is magnified in **f**. Blood cells are autofluorescent (pink). **g** Wild type e14.5 paraffin section, sagittal orientation. **h** *P0-cre; β-cat* ^*fx/fx*^ e14.5 paraffin section, sagittal orientation. **i** & **j** Immunostaining for PDGFRβ (green), counterstained with DAPI (blue) on e17.5 paraffin, coronal sections. **i** Wild type **j** *P0-cre; β-cat* ^*fx/fx*^, blood cells are autofluorescent (pink). All scale bars equal 100 μm

The pattern of *LacZ* expression in the e18.5 *Wnt1-cre; Rosa*^*stopLacZ*^ pituitary suggests that the neural crest contributes to the pituitary vasculature (Fig. 1f). Therefore, we examined the pituitary vasculature in both models of neural crest deficient embryos using immunostaining for CD31 (PECAM), an endothelial cell marker (Fig. 9a–h). The blood vessels in the pituitary glands of both the *Wnt1-cre; β-cat*^*fx/fx*^ and *P0-cre; β-cat*^*fx/fx*^ embryos are abnormally dilated at e18.5 (Fig. 9a–f). Endothelial cells appear to be migrating into the forming pituitary anterior lobe at e14.5 (Fig. 9g). Neither this pattern nor the size of the forming endothelium is disrupted in *P0-cre; β-cat*^*fx/fx*^ embryos, suggesting that the early migration of endothelial cells into the pituitary anterior lobe is unaffected by the loss of the neural crest (Fig. 9h). Pericytes are cells that wrap around the endothelium and regulate blood vessel diameter, and the pericytes of the head are neural crest-derived [17, 18]. Pericytes express PDGFRβ at e18.5 in wild type mouse embryos (Fig. 9i). This pericyte marker is absent in *P0-cre; β-cat*^*fx/fx*^ embryos, confirming that the loss of neural crest cells results in a loss of pituitary gland pericytes (Fig. 9j). *PDGFβ*^*−/−*^ or *PDGFRβ*^*−/−*^ mice develop dilated and leaky vasculature throughout the body that is similar to the vascular defects we observed in the pituitary glands of the *P0-cre; β-cat*^*fx/fx*^ embryos [49–51].

## Discussion

The developing pituitary gland is surrounded by head mesenchyme, which has a dual origin from definitive mesoderm and neural crest [4, 24]. Using two different neural crest expressing transgenic *cre* lines, we have shown that at axial levels the head mesenchyme on the rostral side of Rathke’s pouch is of neural crest origin. This result is in agreement with previous fate mapping experiments of the neural crest derived head mesenchyme [25]. The mesenchyme on the caudal side is derived from definitive mesoderm; the rostral end of the notochord and the prechordal mesoderm is located on the caudal side of Rathke’s pouch [24, 26, 27]. At more lateral levels the boundary between neural crest and definitive mesoderm is still present, but the definitive mesoderm can migrate to the rostral side of Rathke’s pouch. The separation between head mesenchyme of neural crest and definitive mesoderm origins on either side of the pituitary gland presents intriguing possibilities for differential effects of these mesenchymal populations on pituitary gland organogenesis. For instance, stimulation of Notch signaling in the ventral diencephalon reduces *Fgf10* expression in the pituitary organizer, which reduces *Lhx3* expression in Rathke’s pouch and increases apoptosis [52]. *Lhx3* expression is reduced preferentially on the caudal side of Rathke’s pouch and apoptosis is preferentially increased [52]. The differential effects on Rathke’s pouch may be caused by signals from the definitive mesoderm, which are not expressed in the neural crest derived mesoderm.

Mesenchyme is known to be important for pituitary induction [8, 12], but the specific factors involved and source of mesenchyme were unexplored. To address the role of neural crest derived mesenchyme on pituitary organogenesis we examined mouse embryos deficient in neural crest. Intriguingly, ablation of neural crest using two different promoters to drive *cre-*mediated excision of *β-catenin* produced two different pituitary phenotypes. *Wnt1-cre* mediated deletion of *β-catenin* in the neural crest produced Rathke’s pouch and pituitary anterior lobe dysmorphology, without an effect on cell specification, while *P0-cre* mediated deletion had no effect at all, despite equally effective neural crest cell ablation. The dysmorphic pituitary caused by *Wnt1-cre* is likely attributable to expansion of the pituitary organizer, including excess *Bmp4* and *Fgf10* expression, leading to additional oral ectoderm being induced to form Rathke’s pouch. This phenotype is similar to other models of pituitary organizer expansion that result in an enlarged Rathke’s pouch [9, 30–32].

We considered three possibilities for how *Wnt1-cre* mediated inactivation of *β-catenin* could result in a dysmorphic pituitary, and tested these possibilities by using the *Shh-cre* and *P0-cre*. The data from each experiment is summarized in Table 1. The first possibility we considered is that the neural crest pattern the ventral diencephalon, including establishing the rostral boundary of the pituitary organizer, and that neural crest deficient embryos result in a pituitary organizer boundary shift. The neural crest regulate patterning of the forebrain and midbrain [53–55], and could potentially affect patterning in the ventral diencephalon. However, *P0-cre*, which effectively ablates *β-catenin* in the neural crest, does not induce expansion of Rathke’s pouch. Therefore, the loss of neural crest cells is unlikely to cause the expansion of the pituitary organizer.

**Table 1:** Summary of pituitary phenotypes when *β-catenin* is deleted with specific transgenic cre lines

	Efficiency of *β-catenin* deletion in pituitary tissues			
Cre transgene	Neural crest	Ventral diencephalon	Rathke's pouch	Pituitary phenotype
*Wnt1-cre*	Complete	Sporadic	None	Dysmorphic Rathke's pouch and dilated vasculature
*P0-cre*	Complete	None	Sporadic	Normal pituitary morphology and dilated vasculature
*Shh-cre*	None	Sporadic	None	No pituitary phenotype

The second possibility is that modest, ectopic deletion of β-CATENIN in the ventral diencephalon causes expansion of the pituitary organizer. TCF7L2 (TCF4) is a transcription factor that is activated by β-CATENIN, and *Tcf7l2*^*−/−*^ and *Wnt5a*^*−/−*^ embryos both have an expansion of the pituitary organizer that leads to a dysmorphic pituitary [30, 31, 56, 57]. These precedents support the idea that the mosaic deletion of *β-catenin* in a few patches within the ventral diencephalon could be recapitulating the *Wnt5a* and *Tcf7l2* phenotypes. However, *Wnt1-cre* and *Shh-cre* mediate similar mosaic, ectopic β-CATENIN deletion in the ventral diencephalon, but only *Wnt1-cre* causes a phenotype. In addition, *β-catenin* is only ablated in a few cells of this tissue by either *cre* strain. Taken together, these facts argue against the possibility that the pituitary phenotype is caused by deletion of β-CATENIN in the ventral diencephalon.

The third possibility is that *Wnt1-cre* mediated deletion of *β-catenin* in the midbrain results in a rostral shift of the pituitary organizer. The major difference in expression between the *Wnt1-cre* and the *P0-cre* is the expression of the *Wnt1-cre* in the midbrain. Our *Wnt1-cre* fate mapping studies demonstrate that the *Wnt1-cre* is also active in the posterior diencephalon. When *β-catenin* is deleted in the midbrain and posterior diencephalon the patterning of the entire ventral diencephalon is shifted rostrally, including the pituitary organizer, resulting in a dysmorphic Rathke’s pouch. Previous studies have demonstrated that loss of *Wnt1* or *β-catenin* in the mesencephalon causes a loss of midbrain identity and expansion of the hindbrain [19, 58, 59]. *Wnt1* plays a critical role in establishing the isthmic organizer at the boundary between the between the midbrain and hindbrain [60]. Initial characterization of *Wnt1*^*−/−*^ embryos did not identify a disruption in forebrain patterning. Examination of images in a previous characterization of *Wnt1*^*−/−*^ embryos [59], suggests that Rathke’s pouch is expanded in these embryos. Our data indicates that canonical WNT signaling is not only necessary for midbrain-hindbrain boundary formation, but also proper patterning of the ventral diencephalon.

In comparison to the ectodermal structures of the pituitary gland, very little is known about the contributions of the head mesenchyme to pituitary development. Chick explant studies demonstrate a permissive role of mesenchyme in pituitary cell specification, and a potential inductive role of the notochord in Rathke’s pouch formation [8]. A mesenchymal signaling component is also supported by genetic studies in mice, where loss of *Foxd1* in the mesenchyme results in an increase in pituitary anterior lobe cell number and a decrease in LHβ expression [12]. It is not known what the actual mesodermal signal is that regulates pituitary development. The BMP antagonist CHORDIN is expressed in the prechordal mesoderm of mouse embryos, adjacent to the caudal side of Rathke’s pouch [11]. The prechordal plate also expresses NOGGIN, and mice that are *Chrd*^*−/−*^*; Nog*^*+/−*^ develop holoprosencephaly, indicative of midline patterning defects [61]. The *Chrd*^*−/−*^*; Nog*^*+/−*^ embryos do not express *Nkx2.1* in the ventral diencephalon, which leads to a loss of *Fgf8* expression in the pituitary organizer and loss of Rathke’s pouch [61]. These results demonstrate that the prechordal plate is necessary for establishing the pituitary organizer in the ventral diencephalon, which then induces Rathke’s pouch. Direct effects of prechordal plate signaling on development of the pituitary anterior lobe are unknown.

To begin determining what functions the head mesenchyme might play in pituitary organogenesis, we fate mapped the neural crest derivatives near the pituitary gland. Our results unequivocally demonstrate that the neural crest produce the head mesenchyme on the rostral side of Rathke’s pouch, and form a sharp boundary with definitive mesoderm on the caudal side of Rathke’s pouch. While this boundary has been observed surrounding the eye, our results refine the position of the boundary in relation to the pituitary gland with a sharp demarcation occurring at the site of Rathke’s pouch invagination [24]. It is intriguing to speculate that Rathke’s pouch may provide guidance cues to neural crest cell migration. Characterization of the embryonic pituitary transcriptome identified 74 genes with gene ontology (GO) terms related to cell adhesion and migration, including genes with known roles in neural crest migration [62]. Analysis of these candidate genes may reveal differential expression within Rathke’s pouch necessary for directing migrating neural crest cells to the rostral side.

A critical aspect of pituitary development is the delamination of cells from the tightly packed epithelium at the ventral side of Rathke’s pouch, in a rostral direction, as they take on the shape typical of glandular cells, and form the rudimentary anterior lobe. This commences at approximately e12.5 and continues through gestation. Some neural crest derived mesenchyme cells invade the forming anterior lobe at e14.5. This is the same time that vascular invasion is initiated [63–65]. Indeed, at later stages of pituitary development the neural crest derived mesenchyme exhibits a pattern reminiscent of vascular tissue. In the vertebrate head, pericytes, regulatory cells which wrap around endothelial cells, are derived from the neural crest [17, 18]. The neural crest cells invading the anterior lobe are likely forming the pericytes of the pituitary gland. The vasculature of both *Wnt1-cre; β-cat*^*fx/fx*^ and *P0-cre; β-cat*^*fx/fx*^ embryos is dysmorphic and dilated, which is attributable to the lack of regulatory pericytes, as marked by PDGFRβ. Dilated and leaky vasculature results when pericytes are not recruited to the endothelial cells [49, 50]. The vasculature of the *Wnt1-cre; β-cat*^*fx/fx*^ and *P0-cre; β-cat*^*fx/fx*^ pituitaries recapitulates this phenotype and demonstrates a requirement for neural crest derived pericytes in pituitary vasculature formation.

Neither the *Wnt1-cre; β-cat*^*fx/fx*^ or *P0-cre; β-cat*^*fx/fx*^ embryos display a disruption in pituitary anterior lobe cell specification. Therefore, the neural crest derived head mesenchyme is not necessary for normal anterior lobe cell specification. The axial mesoderm on the caudal side of Rathke’s pouch is a rich source for developmental signals, which are known to pattern surrounding tissues, including the neural tube and paraxial mesoderm. Rathke’s pouch sits at a dynamic site at the interface of definitive mesoderm, neural crest, neural ectoderm, and oral ectoderm. Determining the precise molecular mechanisms that each of these tissues plays in pituitary organogenesis in mouse and other vertebrate model systems will help in determining how this vertebrate specific organ may have evolved.

## Conclusions

Using both the *Wnt1-cre* and the *P0-cre* to lineage trace the neural crest, we determined that the mesenchyme on the rostral side of Rathke’s pouch is neural crest in origin. This rostral mesenchyme contributes to the vasculature of the pituitary gland. Deletion of a conditional null allele of *β-catenin* in the neural crest lineage using both the *Wnt1-cre* and *P0-cre* generates mouse embryos that are deficient in neural crest cells [19]. These embryos have dysmorphic pituitary blood vessels and lack PDGFRβ labeled pericytes. The *Wnt1-cre* is also active in the mesencephalon, while the *P0-cre* is not. Loss of *β-catenin* in the mesencephalon causes a loss of midbrain structures [19]. In addition, we have determined that loss of *β-catenin* in the mesencephalon and posterior diencephalon is the likely cause of a rostral shift in the expression domain of *Bmp4* and *Fgf10* in the ventral diencephalon. The altered expression domain of these morphogenetic proteins results in a highly dysmorphic Rathke’s pouch. Therefore, *β-catenin* is required in the *Wnt1* expression domain, including the neural crest, for the proper specification of pituitary gland growth, development, and vascularization.

## Methods

### Mice

The Institutional Committee on the Use and Care of Animals for the University of Michigan and the University of South Carolina approved all experiments using mice (protocol number PRO00004640 at the University of Michigan to SAC and protocol number 2106-100665-012213 at the University of South Carolina to SWD). The *Wnt1-cre*, *Shh -cre*, *β-cat*^*fx/fx*^, *Rosa*^*stopLacZ*^, and *Rosa*^*mT/mG*^ mice were obtained from Jackson Laboratory [19–23]. The *P0-cre* mice were obtained from Dr. Ken-ichi Yamamura [21]. Mice were housed in specific pathogen free conditions with automatic watering, ventilated cages, and fed *ad libitum*. Genotyping of mice was performed as previously reported [19–23]. Observation of copulation plugs in female mice was used to detect mating. Noon of the day the copulation plug was detected was established as e0.5.

### Lineage tracing

β-Galactosidase activity from the *Rosa*^*stopLacZ*^ allele was detected with X-gal staining in cryosections and embryo whole mounts. For cryosections both cre positive and cre negative *Rosa*^*stopLacz*^ embryos were fixed in 4 % paraformaldehyde in LacZ fix for 15 min on ice, washed with LacZ wash, equilibrated in 30 % sucrose, and embedded in O.C.T. Compound (Tissue-Tek) in a dry ice/ethanol bath [66]. Frozen sections were cut on a cryostat at 16 μm, stained with X-gal, and counterstained with Neutral Red [66]. The skin and skull were removed from cre positive and cre negative *Rosa*^*stopLacz*^ embryo whole mounts to allow penetration of X-gal stain. Tissues were fixed for 30 min in 4 % paraformaldehyde in LacZ fix at 4 C, washed three times for 30 min in LacZ wash, and stained with X-gal stain overnight at 37 C. The pituitary was isolated following X-gal staining and photographed on a Leica M125 microscope with a color camera. Fluorescent emission from tdTomato and eGFP was visualized using cre positive and cre negative *Rosa*^*mT/mG*^ embryos on a Leica M125 microscope with fluorescence capabilities and photographed using a Retiga 2000R digital camera.

### Histology, immunohistochemistry, and RNA in situ hybridization

Embryos were fixed overnight in 4 % paraformaldehyde in phosphate buffered saline (PBS) at 4 C, washed and dehydrated, embedded in paraffin, and sectioned at 6 μm [66]. Select sections chosen for histology were stained with hematoxylin and eosin [66].

LHX3 immunofluorescence was performed as previously reported, using a primary antibody dilution of 1:1000 (Developmental Studies Hybridoma Bank, University of Iowa, Iowa City, IA) [30]. Immunofluorescence for β-CATENIN (1:1000, BD Bioscience), CD31 (1:100, PECAM, Thermo Scientific), PDGFRβ (1:100, R&D Systems), OTX2 (1:1000, Abcam), and LEF1 (1:500, Santa Cruz Biotechnology) was performed on paraffin sections. Select sections were processed through Xylene, 100 % ethanol, 95 % ethanol, and PBS, and boiled in 10 mM citric acid for 10 min. Slides used for PECAM, OTX2, and LEF1immunohistochemistry were treated with 1.5 % H_2_O_2_ in 50 % methanol for 20 min. All slides were treated with blocking solution from Perkin-Elmer TSA Tyramide Signal Amplification (TSA) kits before incubating with primary antibodies overnight at 4 C. Species appropriate biotinylated secondary antibodies were used (Jackson ImmunoResearch Laboratories), followed by streptavidin-488 (Jackson ImmunoResearch Laboratories) for PDGFRβ and β-CATENIN or streptavidin-horseradish peroxidase for PECAM, OTX2, and LEF1. PECAM, OTX2, and LEF1 slides were treated with TSA-Fluorescein (Perkin-Elmer, TSA Plus Fluorescein System). A different PECAM antibody (BD Bioscience) and procedure was used for Fig. 8a and b, which was performed on cyrosections as previously reported [67]. Antibodies for the pituitary hormones were obtained from the National Hormone & Peptide Program, and the immunohistochemistry was performed as previously reported, and counterstained with methyl green [68].

RNA in situ hybridization was performed as previously reported for *Six6*, *Bmp4*, and *Fgf10* [9, 28, 69].

### Ethics approval

The Institutional Committee on the Use and Care of Animals for the University of Michigan and the University of South Carolina approved all experiments using mice (protocol number PRO00004640 at the University of Michigan to SAC and protocol number 2106-100665-012213 at the University of South Carolina to SWD).

### Consent for publication

Not applicable.

### Availability of data and material

The data supporting the results of this manuscript are included in the body of the manuscript and as supplemental data.

## Additional files

Additional file 1: Figure S1. a Wild type e18.5 embryo. b *P0-cre; β-cat* ^*fx/fx*^ e18.5 embryo. Scale bars equals 1 mm. (TIF 11308 kb) Additional file 2: Figure S2.
**a** X-gal staining (blue) reveals *Shh-cre* activity in a *Shh-cre; Rosa* ^*stopLacZ*^ e12.5 sagittal section, counterstained with neutral red. **b** Hemotoxylin and eosin staining on e14.5 sagittal sections. **b** Wild type **c**) *Shh-cre; β-cat* ^*fx/fx*^ **d**-**f** Photographs of e14.5 wholemount embryos. **d** Wild type hindlimb, inset shows forelimb. **e** and **f** *Shh-cre; β-cat* ^*fx/fx*^, arrow indicates loss of posterior digits. **e** Hind limb **f** Forelimb **g-i** Immunostaining for β-CATENIN (green) on e14.5 sagittal sections, counterstained with DAPI (blue). **g** Wild type **h** and **i** *Shh-cre; β-cat* ^*fx/fx*^, boxed region in **h** is magnified in **i**. Arrow in **i** indicates a small region with no β-CATENIN expression. Scale bars in **a**–**c** and **g**–**i** equal 100 μm. Scale bars in **d**–**f** equal 1 mm. (TIF 10884 kb)

## Abbreviations

e8.5: embryonic day of development 8.5
DAPI: 4',6-diamidino-2-phenylindole
GH: growth hormone
GO: gene ontology
LH: luteinizing hormone
PBS: phosphate buffered saline
POMC: proopiomelanocortin
*Rosa*^*mT/mG*^: B6.*Gt(ROSA)26Sor*^*tm4(ACTB-tdTomato,-EGFP)Luo*^
*Rosa*^*stopLacZ*^: *Gt(ROSA)26Sor*^*tm1Sho*^
*Shh-cre*: *Shh*^*tm1(EGFP/cre)Cjt*^
TSA: tyramide signal amplification
TSH: thyroid stimulating hormone
*Wnt1-cre*: *Tg(Wnt1-cre)11Rth*
*β-cat*^*fx/fx*^: *Ctnnb1*^*tm2.1Kem*^
