# Supplementary figures and images for: β-catenin is required in the neural crest and mesencephalon for pituitary gland organogenesis
Source: BMC Dev Biol. 2016 May 16;16:16. doi: 10.1186/s12861-016-0118-9 (PMC4868042; doi:10.1186/s12861-016-0118-9)

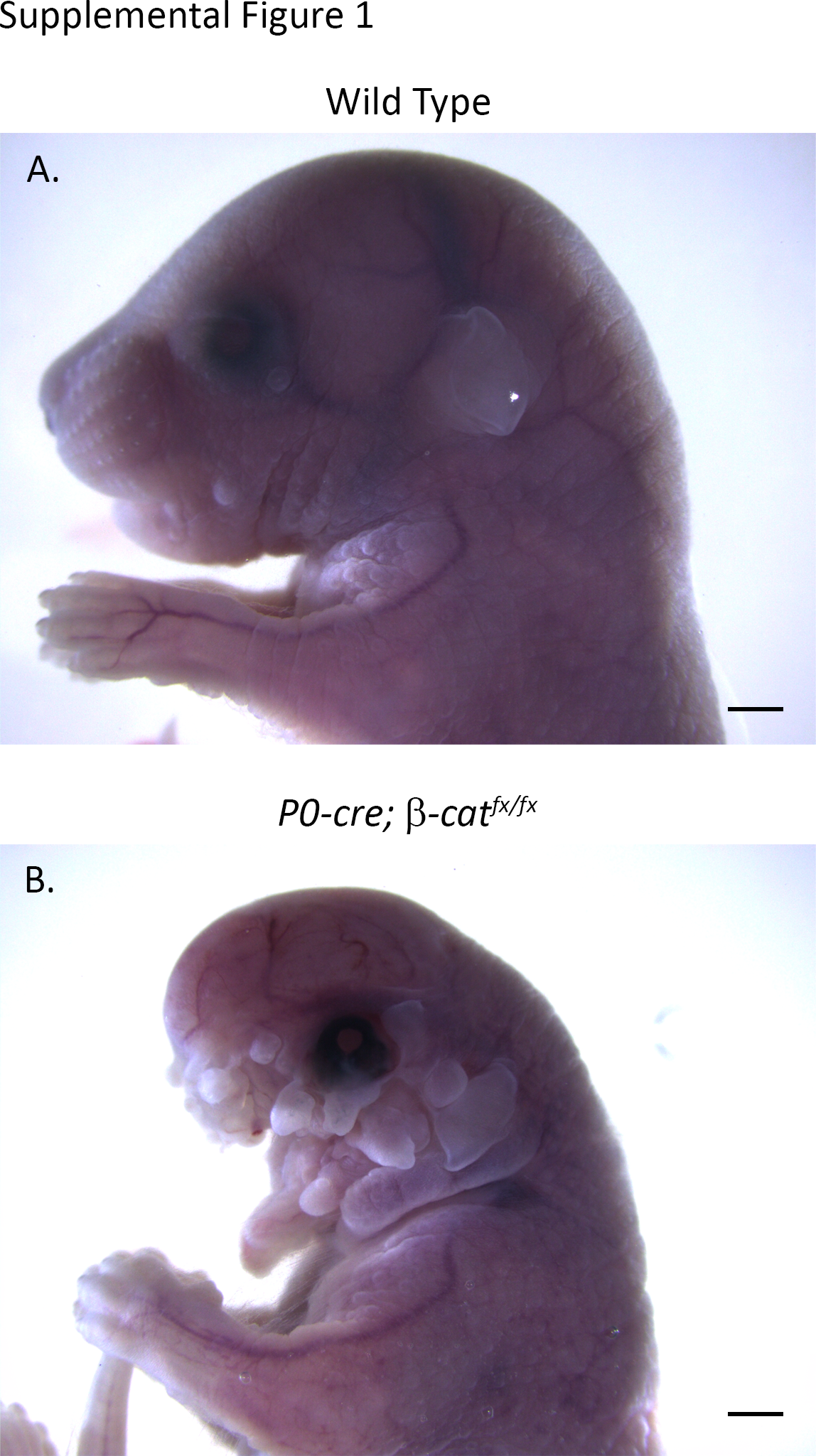

Supplement: Additional file 1: Figure S1. — a Wild type e18.5 embryo. b P0-cre; β-cat fx/fx e18.5 embryo. Scale bars equals 1 mm. (TIF 11308 kb) [file 12861_2016_118_MOESM1_ESM.tif]

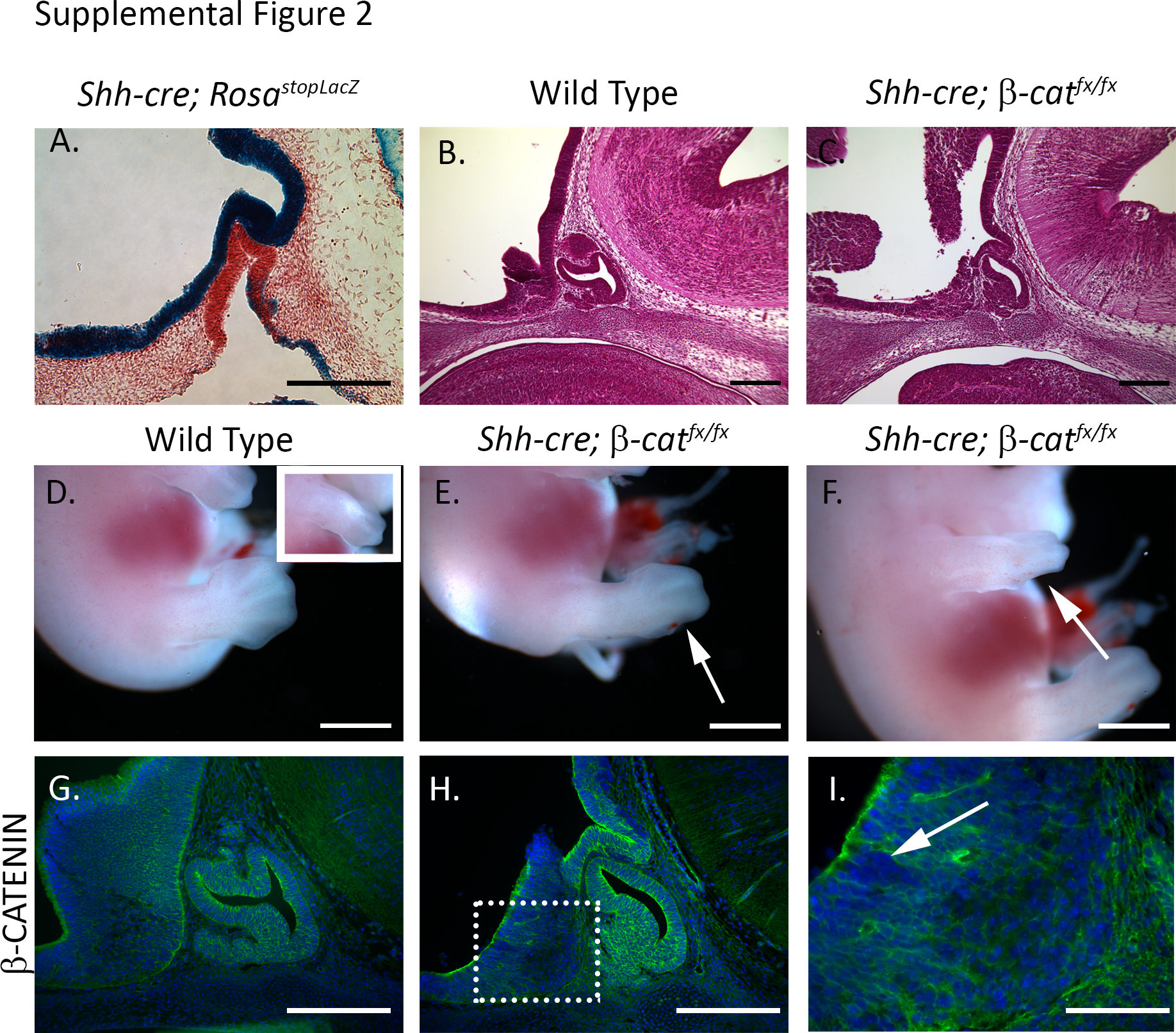

Supplement: Additional file 2: Figure S2. — a X-gal staining (blue) reveals Shh-cre activity in a Shh-cre; Rosa stopLacZ e12.5 sagittal section, counterstained with neutral red. b Hemotoxylin and eosin staining on e14.5 sagittal sections. b Wild type c) Shh-cre; β-cat fx/fx d-f Photographs of e14.5 wholemount embryos. d Wild type hindlimb, inset shows forelimb. e and f Shh-cre; β-cat fx/fx, arrow indicates loss of posterior digits. e Hind limb f Forelimb g-i Immunostaining for β-CATENIN (green) on e14.5 sagittal sections, counterstained with DAPI (blue). g Wild type h and i Shh-cre; β-cat fx/fx, boxed region in h is magnified in i. Arrow in i indicates a small region with no β-CATENIN expression. Scale bars in a–c and g–i equal 100 μm. Scale bars in d–f equal 1 mm. (TIF 10884 kb) [file 12861_2016_118_MOESM2_ESM.tif]
